# The Distance between N and C Termini of Tau and of FTDP-17 Mutants Is Modulated by Microtubule Interactions in Living Cells

**DOI:** 10.3389/fnmol.2017.00210

**Published:** 2017-06-30

**Authors:** Cristina Di Primio, Valentina Quercioli, Giacomo Siano, Matteo Rovere, Branislav Kovacech, Michal Novak, Antonino Cattaneo

**Affiliations:** ^1^Bio@SNS Laboratory, Scuola Normale SuperiorePisa, Italy; ^2^Institute of Neuroimmunology, Slovak Academy of Sciences, Axon Neuroscience SEBratislava, Slovakia

**Keywords:** Tau, biosensor, conformation, FRET, FRAP, mutation

## Abstract

The microtubule (MT)-associated protein Tau is a natively unfolded protein, involved in a number of neurodegenerative disorders, collectively called tauopathies, aggregating in neurofibrillary tangles (NFT). It is an open question how the conversion from a MT bound molecule to an aggregation-prone Tau species occurs and, also, if and how tauopathy-related mutations affect its behavior in the cell. To address these points, we exploited a genetically encoded FRET sensor based on the full length Tau protein, to monitor in real time Tau conformational changes in different conditions in live cells. By studying the FRET signal we found that soluble Tau molecules, detached from MTs, display an unfolded structure. On the contrary, we observed an increased FRET signal generated by Tau monomers bound to MT, indicating that the association with MTs induced a folding of Tau protein, decreasing the distance between its N and C termini. We exploited the FRET sensor to investigate the impact of FTDP-17 mutations and of phosphorylation-site mutations on Tau folding and mobility in live cells. We demonstrated that the FTDP-17 Tau mutations weaken the interaction of Tau with cellular MTs, shifting the equilibrium towards the soluble pool while, conversely, phosphorylation site mutations shift the equilibrium of Tau towards the MT-bound state and a more closed conformation.

## Introduction

The microtubule (MT) associated protein Tau (Goedert et al., 1989a,b) plays a central role in neuronal cell biology, since it maintains the cytoskeleton stability, promotes axonal outgrowth and regulates the axonal trafficking (Arendt et al., 2016).

During neurodegeneration, Tau undergoes post-translational modifications, truncations and aggregation, and has been identified as the main component of neurofibrillary tangles common in AD and other tauopathies (Goedert et al., 1988; Kondo et al., 1988; Wischik et al., 1988a,b; Kovacech and Novak, 2010; Iqbal et al., 2013; Falcon et al., 2015; Min et al., 2015; Houck et al., 2016; Wang and Mandelkow, 2016).

Biophysical characterizations demonstrated that Tau is an intrinsically disordered protein and, as other natively unfolded proteins, it tends to be highly flexible and to have variable conformations *in vitro* (Wright and Dyson, 2015). A number of reports have shown that unstructured proteins can acquire distinct important biological functions when they interact with other binding partners (Dunker et al., 2002; Tompa, 2002; Uversky, 2002; Wright and Dyson, 2015).

Tau interaction with microtubules via the Microtubules Binding Repeat Domains (MBRD) is fundamental for the maintenance of cytoskeleton stability and several mutations in the MBRD lead to pathology (Barghorn et al., 2005; Akoury et al., 2013). Several point mutations are responsible for an early form of dementia called FTDP-17, characterized by Tau aggregates (Nacharaju et al., 1999; Vogelsberg-Ragaglia et al., 2000; Spillantini et al., 2006). Functional assays of Tau proteins with different FTDP-17 mutations demonstrated the alteration in binding microtubules and in promoting MT assembly (Hong et al., 1998; Nagiec et al., 2001; Krishnamurthy and Johnson, 2004; Goedert and Jakes, 2005; Fischer et al., 2007).

However, the mode of action of Tau and the modifications and mutations inducing its pathological behavior are still enigmatic. Two hypotheses have been proposed: Tau phosphorylation renders Tau unable to bind microtubules triggering MTs destabilization, or MTs depolymerization increases the pool of soluble Tau that is a good substrate for further phosphorylation (Cash et al., 2003; Jope and Johnson, 2004; Hernandez et al., 2013).

Despite a number of biophysical structural studies on Tau, or Tau fragments, *in vitro* (Woody et al., 1983; Wischik et al., 1988a,b; Hasegawa et al., 1998; Esposito et al., 2000; Barghorn et al., 2005; Kuret et al., 2005; Kadavath et al., 2015a,b) little is known about full length Tau conformations in the presence or absence of MTs in the context of live cells.

Here we describe a genetically encoded Foster Resonance Energy Transfer (FRET) sensor that is sensitive to Tau conformation in the cell context.

The Conformational Sensitive Tau sensor (CST) is based on Tau full length protein and allows monitoring the conformation of molecules interacting or not with cellular MTs, in live cells treated with drugs depolymerizing or stabilizing microtubules. We exploited this tool to study the impact of the P301L and ∆K280 mutations involved in FTDP-17, as well as of another phosphorylation-defective Tau mutant (altering Tau interactions with MT, Tau conformation and mobility).

## Materials and Methods

### Chimeric Constructs Cloning

The cDNA encoding the Tau isoform D (383aa) has been cloned into the BspEI site of the plasmid pECFP-EYFP already available in the lab. Both the forward and reverse cloning primers contain the RSIVT linker sequence between the BspEI site and the Tau sequence (forward primer: 5′-GTCGTT TCCGGAAGATCTATTGTCACTATGGCTGAG-3′; reverse primer: 5′-AACGACTCCGGA AGTGACAATA- GATCTCAAACCCTG-3′). The monolabeled constructs pECFP-Tau and pTau-EYFP have been generated by subcloning the Tau cDNA into the BspEI site of plasmid pECFP-C1 and pEYFP-N1 (Clontech Laboratories, Inc., Saint-Germain-en-Laye, France) (FWD-BspEI-TAU: 5′-AATTATT- CCGGAATGGCTGAGCCCACGCCAG-3′; REV-BspEI-TAU: 5′-ACTTGATCCGGACAAACCCTGCTTAGGCCAG-3′; FWD- BspEI-RSIVT-TAU: 5′-AATTATTCCGGAAGATCTATTGT CACTAT GGCTGAGCCCACGCCAG-3′; REV-BspEI-RSIVT-TAU:5′-ACTTGAT CCGGAAGTGACAATAGATCTC AAA CCCTGCTTAGGCCAG-3′). The CST mutants have been generated by the Q5 Site-Directed Mutagenesis Kit (New England BioLabs) (CST-AT8mut primers to introduce the mutations S199A-S202A-T205A: Fwd 5′-CCCA GGCGCACCCGGCAGCCGCTCCCGC-3′; Rev5′-GCG CCGGGGGCGCTGTAGCCGCTGCGATCCCC-3′;CST-P301L primers: Fwd 5′-AAACACGTCCTGGGAGGCGGC-3′; Rev 5′-GATATTATCCTTTGAGCCACACTTGGACTG-3′; CST-∆K280 primers: Fwd 5′-AAGCTGGATCTTAGCAAC-3′; Rev 5′-ATTAATTATCTGCACCTTCC-3′). The pTagRFP-tubulin plasmid was purchased from Evrogen (FP145).

### Cell Culture, Transfection and Treatments

HeLa cells and immortalized hippocampal neurons HT22 were maintained in DMEM (GIBCO) supplemented with 10% FCS. The day before the experiment cells were seeded at 10 × 10^4^ cells per well in six-well plates or in Willco dishes (Willcowells). The lipofection was carried out with Effectene (QIAGEN) according to manufacturer’s instructions. Cells have been treated with 1 μM Nocodazole (Sigma) for 30 min, or with 1 μM Paclitaxel (SIGMA) for 10 min. Tau seeds have been produced by *in vitro* oligomerization. The oligomerization reaction has been performed with 4 mg/ml truncated Tau (297-391)4R in PBS pH 7.2 and 100 μM Heparin (Sigma, H3149) and incubated for 4 days at room temperature. The seeds were purified by centrifugation at 100,000× *g* for 1.5 h, the pellet was rinsed with PBS. Microtubules labeling in live cells has been obtained by SiR-tubulin Kit (excitation wavelength *λ* = 633 nm) (SPIROCHROME) in FRET experiments to avoid interference in FRET signal.

### Western Blot and Immunostaining

For Western blot cells extracts were prepared in lysis buffer supplemented with protease and phosphatase inhibitors by lysis on ice for 30 min. Total proteins were separated by 10% or 8% SDS-PAGE and electro-blotted onto nitrocellulose membranes Hybond-C-Extra (Amersham Biosciences). Membranes were blocked with 5% skimmed milk powder in TBS containing 0.1% Tween 20. For the immunofluorescence cells were fixed with ice-cold 100% methanol × for 5 min. After permeabilization with PBS 1× containing 0.1% Triton-X100 for 10 min, samples were blocked for 1 h with 1% (wt/vol) BSA at RT. The slides were incubated with the primary antibody 1 h at room temperature and with secondary antibodies fluorophore-conjugated 1 h at room temperature. Slides were mounted with Vectashield mounting medium (Vector Laboratories). Primary antibodies were: mouse monoclonal anti-tau (Tau5) ab80579 (abcam); mouse monoclonal anti-a-Tubulin Clone B-5-1-2 (SIGMA-ALDRICH); mouse monoclonal anti-GAPDH (Fitzgerald); mouse monoclonal anti-Tau DC25 (from Novak M.); mouse monoclonal anti-Tau Clone AT8 (MN1020, Thermo Scientific). Secondary antibodies for Western blot analysis were HRP-conjugated anti-mouse or anti-rabbit, purchased from Santa Cruz Biotechnology, Inc., Santa Cruz, CA, USA. Secondary antibodies for IF: Alexa Fluor 633 rabbit anti-mouse IgG (Life Technologies).

### Image Acquisition and Analysis

Images were acquired with the TCS SL laser-scanning confocal microscope (Leica Microsystems, Milan, Italy) equipped with galvanometric stage using a 63×/1.4 NA HCX PL APO oil immersion objective. A heated and humidified chamber mounted on the stage of the microscope was used for live imaging experiments in order to maintain a controlled temperature (37°C) and CO_2_ (5%) during image acquisition. An Argon laser was used for ECFP (*λ* = 458 nm) and EYFP (*λ* = 514 nm), a Gre-Ne laser for RFP (*λ* = 543 nm) and a He-Ne laser for *λ* = 633 nm. For the quantification of morphological parameters such as the total filament length and the number of crossover points the filament tracer option of the IMARIS Bitplane software has been exploited. In detail, these two parameters are deduced by a software plugin that, based on connectivity and fluorescence intensity, automatically detects and segments filamentous structures revealing information about the topology of filaments as the sum of the lengths of all lines and the number of crossover points within the filament.

### FRET and FRAP Experiments

For sensitized emission FRET experiments, each image was recorded in a spectral mode, by selecting specific regions of the emission spectrum. The donor ECFP was excited at 458 nm and its fluorescence emission was collected between 470 nm and 500 nm (donor channel) and between 530 nm and 600 nm (FRET channel). The acceptor EYFP was excited at 514 nm and its fluorescence emission was collected between 530 nm and 600 nm (acceptor channel). The donor and acceptor fluorophores were excited sequentially. The ImageJ software was used for images analysis and FRET quantification. Briefly, FRET images were corrected from cross-talk between donor and acceptor channel using Youvan’s method (Youvan et al., 1997): F_index_ = I_FRET_−A × I_D_−B × I_A_, where I_FRET_, I_D_ and I_A_ are the images of the sample in the FRET, donor and acceptor channel, respectively, after background subtraction and A and B are the fraction of the donor and acceptor leak-through into the FRET channel, respectively. We determined the A parameter in cells expressing only the donor (pECFP plasmid) by acquiring images in donor and FRET channels. A plot of the intensity of each pixel of the FRET channel image as a function of the intensity of the same pixel in the donor channel image (regression graph) was automatically generated by the ImageJ plugin (FRET and colocalization Analyzer) and then fitted with a linear equation from which A parameter was derived. Images displaying saturated pixel has been discarded. For the evaluation of the B parameter cells transiently expressing only the acceptor (pEYFP plasmid) have been acquired in FRET and acceptor channel and then subjected to same process of A in the ImageJ plugin. Typical values in our experimental conditions are *A* = 0.1 and *B* = 0.25, respectively. Normalized FRET (NFRET) was performed with the ImageJ software plugin “pixFRET” (Feige et al., 2005) by using: NFRET = F_index_/√(I_D_ × I_A_) (Xia and Liu, 2001). Mathematically, NFRET values should range between 0–1; the plugin automatically multiplies these values ×100; NFRET intensities images were represented in false-color obtained by using the “fire” lookup table option in ImageJ software and shown from 0 to 60. We setup this color scale in order to better appreciate also lower NFRET signals. Due to the mathematical subtractions in F_index_ calculation, NFRET negative values in some pixels are considered as zero. In FRET experiments investigating intramolecular and inter-molecular FRET, to obtain a comparable amount of Tau we transfected cells with 300 ng of CST or 150 ng ECFP-Tau + 150 ng EYFP-Tau (ML/2); to obtain a comparable amount of fluorophores we transfected cells with 300 ng ECFP-Tau + 300 ng EYFP-Tau (ML). Since in a transient transfection the expression of the transgene varies from cell to cell, to perform a reliable NFRET quantification in ML samples we selected cells that express comparable amount of fluorescence intensity in the donor and acceptor channel with respect of CST; to perform the NFRET quantification in ML/2 samples we selected cells that express about half the amount of fluorescence intensity in the donor and acceptor channel with respect of CST. We measured the level of expression by fluorescence intensity in individual cells, under the microscope. FRAP experiments were performed by using the FRAP module coupled to the confocal microscope and consists of three different phases: (1) a pre-bleach phase, in which 10 frames of 512 * 512 pixel images at 1000 Hz have been recorded in order to define the initial level of fluorescence intensity; (2) a photobleaching phase, in which a selected circular ROI with a radius of 2 μm in the cytoplasm of the cell was excited at higher laser power (50% for EYFP) for five frames at 1000 Hz; (3) a post-bleaching phase, in which 120 images have been recorded in order to follow the recovery of the fluorescence intensity in the selected ROI. Fluorescence recovery was extracted from images of the bleached ROI and subjected to the following manipulation steps: (1) background subtraction; (2) first normalization to the initial pre-bleach value of fluorescence intensity; (3) correction for the fluorescence loss: in particular, we evaluated the fluorescence of the whole cell for each time point, before and after the photobleaching phase; then we multiplied every element of the data of the bleached ROI by (F_pre-wholecell_/F_post-wholecell_), where F_pre-wholecell_ means prebleach intensity within the whole cell, F_post-wholecell_ stands for postbleach intensity at any given time point. In any case, we set up the experimental parameters of acquisition in order to avoid strong photobleaching (5%–10%); (4) additional normalization to set the first post-bleach point to zero. At least 30 separate FRAP experiments for each sample has been performed. FRAP recovery curves have been fitted by a two phase exponential association function (OriginLab): *y* = *y*_0_ + *A*_1_^*^ × (1 − exp(−*t*/*τ*_1_)) + *A*_2_^*^ × (1 − exp(−*t*/*τ*_2_)); fractions *A*_1_ and *A*_2_ reported in the Supplementary Table S1 are defined as: *A*_1_ = *A*_1_^*^/(*A*_1_^*^ + *A*_2_^*^) and A_2_ = *A*_2_^*^/(*A*_1_^*^ + *A*_2_^*^); the mobile fraction is defined as Mob_calc_ = *A*_1_^*^ + *A*_2_^*^.

## Results

### Microtubule-Bound Tau Molecules Display a Hairpin Conformation

**Figure 1:**
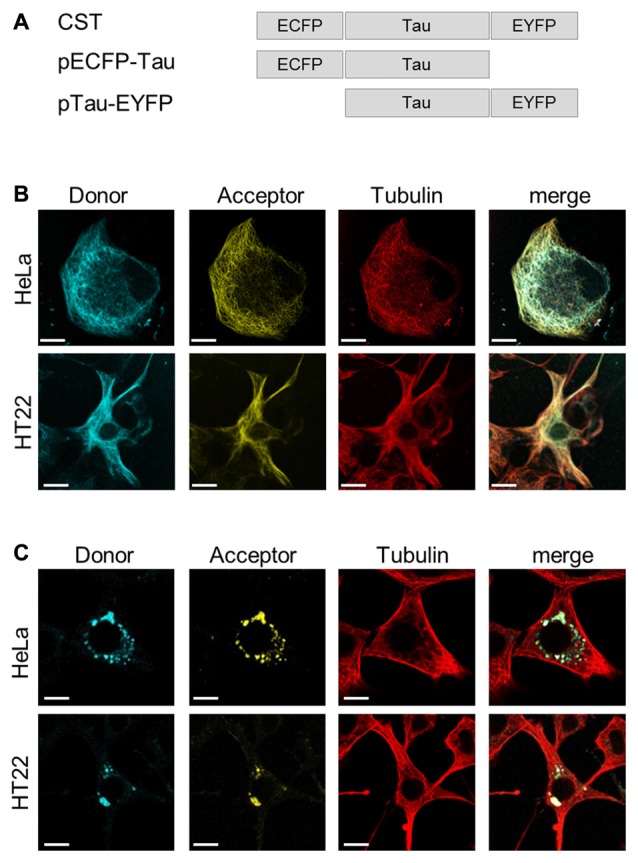
ConformationalSensitive Tau sensor (CST) construct and imaging in live cells. **(A)** Schematic representation of chimeric constructs. **(B)** Imaging of HeLa and HT22 cells co-transfected with CST and Tubulin-RFP plasmids; donor channel (blue), acceptor channel (yellow), Tubulin (red). White scale bar = 10 μm. **(C)** Imaging of Hela and HT22 cells 48 h after treatment with synthetic Tau seeds; donor channel (blue), acceptor channel (yellow), Tubulin (red). White scale bar = 10 μm.

To investigate whether, in physiological conditions, the binding to MTs induces Tau protein folding rearrangements, we exploited a conformational sensitive fluorescent sensor by fusing ECFP at the N-terminus and EYFP at the C-terminus of the full length human Tau-D sequence (0N4R) (Figure 1A). It is expected that, depending on the conformation of Tau, a FRET signal could be generated by the decreased distance between the N- and C-termini of Tau molecules, thus realizing a context-dependent Conformational-Sensitive Tau sensor (CST). Mono-labeled fluorescent Tau constructs have been used as controls.

Confocal microscopy imaging in live cells showed that CST decorated the cellular MTs network and colocalized with the tubulin-RFP, both in HeLa cells, that do not express endogenous Tau, and in immortalized hippocampal neurons HT22 (Figure 1B), demonstrating that, under physiological conditions, the CST preserves Tau ability to interact with MTs.

Current views indicate that misfolded Tau drives its own aggregation and spreading in Tauopathy disease progression (Frost et al., 2009; Kfoury et al., 2012; Le et al., 2012; Holmes and Diamond, 2014; Holmes et al., 2014; Mirbaha et al., 2015).

To verify whether CST could detect the seeding induced by molecules potentially involved in pathological self-aggregation, reporter cells expressing CST were exposed to extracellular synthetic Tau seeds, prepared from (297-391)4R recombinant Tau fragment (Kontsekova et al., 2014). Forty-eight hours later the Tau network decorating MTs almost disappeared and concomitantly fluorescent cytoplasmic inclusions appeared (Figure 1C), while the MT network was not affected.

Altogether, these results indicated that CST is sensitive to Tau seeding activities and it allows detecting the Tau displacement from MTs and its aggregation.

**Figure 2:**
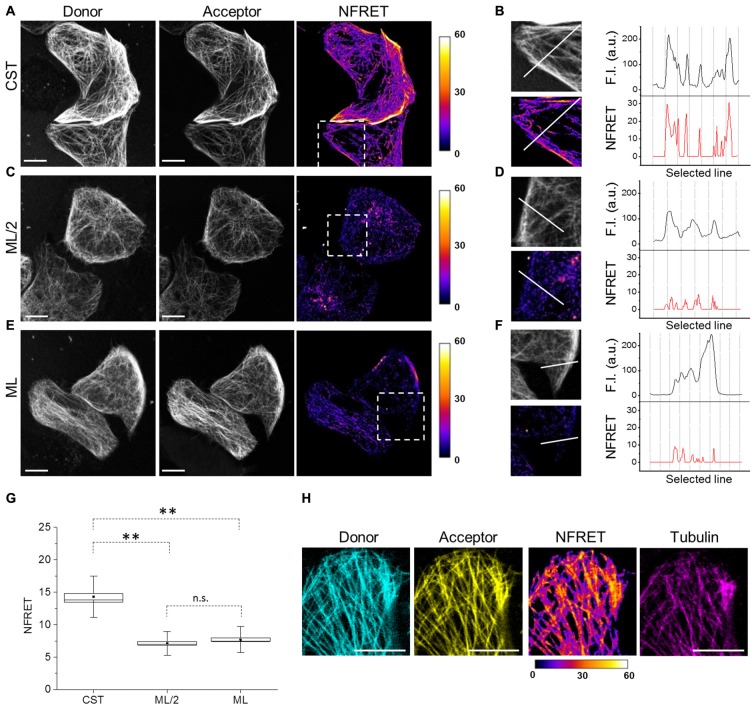
Tau conformation on microtubules (MTs) by Foster Resonance Energy Transfer (FRET). **(A)** FRET measurements in CST reporter HeLa cells. Donor (gray), acceptor (gray) and Normalized FRET (NFRET) images (false color). White scale bar = 10 μm. **(B)** Magnification of the outlined box in **(A)**. Fluorescence intensity profile (F.I.) along the white line (black graph) and NFRET intensity profile along the white line (red graph). **(C)** FRET measurements in cells expressing ECFP-Tau and Tau-EYFP at comparable Tau levels to CST (ML/2). **(D)** Magnification of the outlined box in **(C)**. F.I. along the white line (black graph) and NFRET intensity profile along the same white line (red graph). **(E)** FRET measurements in cells co-expressing monolabeled ECFP-Tau and Tau-EYFP at comparable fluorophore levels to CST (ML). **(F)** Magnification of the outlined box in **(G)**. F.I. along the white line (black graph) and NFRET intensity profile along the white line (red graph). **(G)** Box plot of NFRET values calculated on MTs network in CST (40 cells), ML/2 (50 cells) and ML (60 cells). Box spans the standard error of the mean (black square) while whiskers indicates the standard deviation (**p < 0.001 ANOVA one-way test). **(H)** NFRET signal localization along MTs. Donor (blue), acceptor (yellow), NFRET (false color) and tubulin labeled with Sir-tubulin (magenta); Pearson coefficient_donor/Tubulin_: 0.797; Pearson coefficient_acceptor/Tubulin_: 0.839; Pearson coefficient_NFRET/Tubulin_: 0.742. White scale bar = 10 μm.

To exploit the CST as a conformational probe, we used quantitative sensitized emission FRET microscopy for the detection of protein conformational changes. First, we set FRET parameters in cells expressing the donor and the acceptor fused into a chimeric construct (ECFP-EYFP), as a FRET positive control, and in cells expressing the donor and the acceptor from separate plasmids (pECFP and pEYFP), as a FRET negative control (Supplementary Figure S1). Figure 2A shows donor and acceptor imaging of CST and also a NFRET image (Feige et al., 2005), indicating that CST displayed a FRET-positive signal mostly on MT lattice. Indeed, along selected lines (white line Figure 2B) at the intersection with MTs, the CST displayed NFRET values in the range of 15–30.

To verify whether the observed FRET signal was due to intramolecular or to intermolecular interactions between the Tau N- and C-termini, cells co-expressing monolabeled Tau constructs (ECFP-Tau and Tau-EYFP) have been analyzed. Due to the presence of two fluorophores per Tau molecule in the CST construct, we compared the FRET signals obtained from CST expressing cells, with that obtained from cells expressing monolabeled Tau. We considered either cells expressing the same levels of monolabeled Tau, with respect to CST cells (hence half amounts of fluorophores, ML/2; Figures 2C,D) or cells expressing double levels of monolabeled Tau, with respect to CST cell (hence same amount of fluorophores, ML; Figures 2E,F). In both conditions, the NFRET signal from the cells expressing monolabeled Tau was significantly lower than that observed with CST (Figures 2D,F). The comparative quantification of the FRET values shows a statistically significant difference between the various conditions (Figure 2G).

These results demonstrate that MT-bound CST assumes a hairpin three-dimensional conformation, with its N-terminal and C-terminal domains brought in proximity, allowing FRET to occur. On the contrary, in conditions where only intermolecular FRET would occur, such as when monolabeled Tau fluorescent pairs are expressed, the FRET signal is significantly lower. The FRET signal shows a precise co-localization with the MT network, as revealed by incorporation of fluorescent tubulin (Sir-tubulin) in cellular MTs (Figure 2H), thus demonstrating that the FRET signal arose indeed from a conformation of the CST upon binding to MTs.

**Figure 3:**
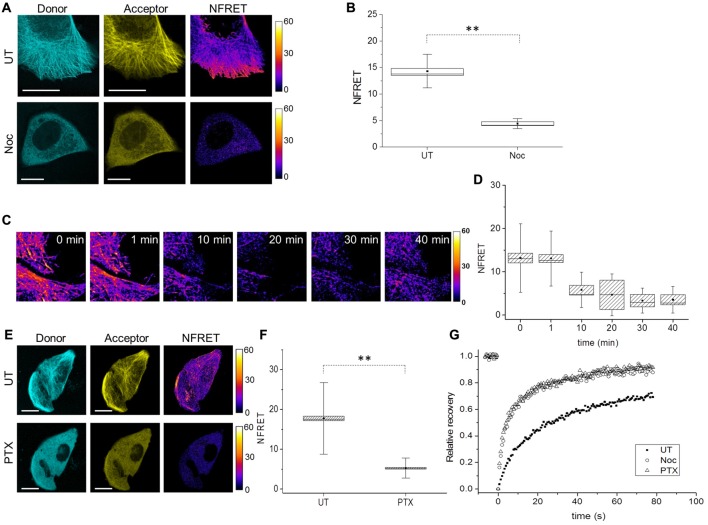
Tau conformation in cells treated with MT interfering drugs analyzed by FRET. **(A)** Donor (blue), acceptor (yellow) and NFRET images (false color) in CST reporter cells untreated (UT, upper panels) or treated with Nocodazole (Noc, lower panels). White scale bar = 10 μm. **(B)** Box plot of NFRET values in untreated cells (40 cells) along MTs and in Nocodazole treated cells (20 cells) in random points in the cytoplasm. Box spans the standard error of the mean (black square) while whiskers indicates the standard deviation (**p < 0.001 ANOVA one-way test). **(C)** Real-time FRET analysis of Nocodazole treated reporting cells. NFRET is displayed in false color. **(D)** NFRET quantification of C at different time points. **(E)** Donor (blue), acceptor (yellow) and NFRET images (false color) in CST reporter cells untreated (UT, upper panels) or treated with Paclitaxel (PTX, lower panels). White scale bar = 10 μm. **(F)** NFRET quantification of **(E)** along MTs in untreated cells or in random points in PTX treated cells. Box spans the standard error of the mean (black square) while whiskers indicates the standard deviation (**p < 0.001 ANOVA one-way test). **(G)** FRAP relative recovery curves of CST in untreated cells (black square), treated with Nocodazole (empty circle) or Paclitaxel (empty triangle).

To analyze the conformation of soluble Tau monomers, not bound to MTs, CST reporter cells were treated with the MTs disrupting drug Nocodazole (Noc). In this condition, the CST diffused into the cytoplasm (Figure 3A) and concomitantly, the FRET signal was lost (Figures 3A,B), indicating that its N- and C-termini are not close one to another, in this condition. It is noteworthy that the CST rendered soluble by depolymerizing MTs does not show signs of aggregation. Western blot analysis showed that, under these conditions, most of the CST molecules are intact (Supplementary Figure S2A), thus the lack of FRET signal is not due to the cleavage of fluorophore moieties.

A time course analysis of the FRET signal, as a function of the time of incubation of cells with nocodazole showed that the loss of FRET signal parallels the MT disruption (Figures 3C,D), and that just 10 min after addition of nocodazole, MTs start to disassemble and NFRET dropped by almost 50%. This further demonstrates that CST can be used to monitor the state of association of Tau to cellular MTs.

We sought to determine whether loss of FRET signal is a consequence of MT disruption or only of the detachment of Tau from MTs, into a soluble pool. To this aim we exploited the MT stabilizing drug Paclitaxel (PTX), that while inducing a stabilization of MTs, competes with Tau for its binding to MTs (Kar et al., 2003; Makrides et al., 2004; Hernández et al., 2013). CST expressing cells were PTX treated for 10 min, after which cells were imaged for Tubulin immunofluorescence and CST fluorescence (Supplementary Figures S2B,C), showing that while the MT network is intact, Tau does no longer decorate MTs. Moreover, the CST is not processed in PTX treated cells (Supplementary Figure S2C). Under these conditions, no FRET signal can be observed (Figures 3E,F).

Next, we investigated Tau mobility by Fluorescence Recovery After Photobleaching (FRAP). FRAP technique allows to distinguish the freely diffusing pool of proteins, compared to the pool bound to other structures (Azoury et al., 2008; Himmel et al., 2009). The rate of fluorescence recovery indicates how fast neighboring fluorescent molecules fill the bleached area and the protein mobility is determined by both diffusion and binding interactions (Breuzard et al., 2013). We performed FRAP experiments in CST reporter cells, either untreated, or treated with Nocodazole or PTX. The corresponding recovery curves were fitted with two-exponential functions (Figure 3G, Supplementary Table S1 and Supplementary Figure S2D) and show that the CST FRAP curves in the Nocodazole and the PTX treated cells are identical, and different from the corresponding curve from untreated cells (Figure 3G).

We observed that 76% ± 3 of the CST molecules in untreated cells are in the mobile fraction contributing to fill the bleached area (Supplementary Table S1). The recovery curve analysis revealed that two main components are represented in this mobile fraction: the 27.8% ± 4.1, ascribed to diffusing soluble molecules (*A*_1_) and the 72% ± 5, ascribed to a MTs-bound phase (*A*_2_). The time constant associated with the rapid diffusion component is *τ*_1_ of 4.9 ± 0.6 s while the fluorescence recovery due to MT-bound CST molecules occurred with a time constant which was seven-fold longer (*τ*_2_ = 35.2 ± 2.8 s. These values are in agreement with previous studies, considering the presence of two fluorophores in each CST molecule (Breuzard et al., 2013).

The corresponding values for the Nocodazole and the PTX-treated cells show a large increase in the overall mobile fraction (91 ± 4 and 93 ± 3 respectively), with a two-fold increase in the diffusive fraction (53 ± 5 and 55 ± 3 respectively) and the concomitant reduction of the MT-bound fraction (47 ± 4 and 45 ± 3 respectively). The fact that we still observe two components with Nocodazole, while Breuzard et al. (2013) fit their FRAP Nocodazole curves with only one exponential might be due to incomplete disruption of the MT network, under our experimental conditions. These results provide a further demonstration that the Tau conformation in the soluble pool does not depend on the integrity of the MT network, but is rather an intrinsic property of soluble Tau molecules.

In conclusion, these results demonstrate that, in the cell, Tau molecule bound to MTs assumes a structured hairpin folding conformation, while it is an unstructured protein when not bound to MTs.

### FTDP-17 Point Mutations Alter Tau Conformation and Mobility

**Figure 4:**
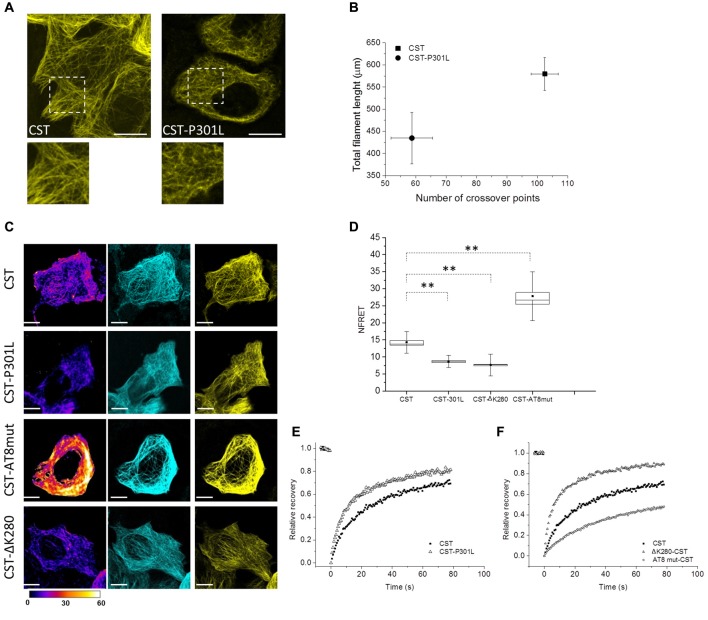
Impact of different point mutations on Tau conformation and mobility. **(A)** Confocal imaging of reporter cells (Acceptor channel, yellow) expressing CST and CST-P301L. White boxes are image magnifications. **(B)** Morphological analysis of fluorescent network density in reporter cells expressing CST (*n* = 10), CST-P301L (*n* = 10). Data shown are mean and SEM. **(C)** NFRET images (false color), donor (Blue), acceptor (yellow) of reporter cells. White scale bar = 10 μm. **(D)** NFRET quantification along MTs of cells expressing CST (*n* = 40), CST-P301L (*n* = 35), CST-∆K280 (*n* = 20) and CST-AT8mut (*n* = 20). Box spans the standard error of the mean (black square) while whiskers indicates the standard deviation (**p < 0.001 ANOVA one-way test). **(E)** FRAP relative recovery curves of CST and CST-P301L. **(F)** FRAP relative recovery curves of CST, CST-∆K280 and CST-AT8mut.

Tau point mutation at residue 301 is involved in FTDP-17 and is known to promote self-aggregation, but its contribution to the conformation of full length protein and to its mobility has not been clarified yet. Reporter cells expressing CST bearing P301L mutation have been analyzed by image analysis, FRET and FRAP (Figure 4). Fluorescence confocal imaging of CST-P301L reporter cells revealed a remarkable qualitative difference in MTs decoration by this Tau mutant, compared to CST, highlighted by magnification boxes (Figure 4A).

Image processing and analysis quantified the total length of Tau labeled segments on MTs and the number of crossover points of the fluorescent network (Figure 4B and Supplementary Figure S3D). Cells with comparable fluorescence intensity were analyzed. The results show, for both parameters, a significant decrease in the cells expressing the CST-P301L. This analysis indicates, for Leu mutant, an impaired ability of interacting with MTs. Conversely, the expression of these mutants does not affect the overall architecture of the MT lattice, nor the integrity and stability of the CST (Supplementary Figure S3A).

FRET analysis clearly demonstrated that P301 mutation altered Tau conformation on MTs, reducing by 50% the NFRET value (from 14.5 ± 0.5 to 8 ± 0.3; Figures 4C,D). This result indicates that P301L Tau mutant, even when it is associated to MTs, has an extended conformation that disadvantage FRET events.

Both image analysis and FRET experiments indicated that the P301L mutation strongly interferes with protein conformation and MTs interplay, therefore we investigated Tau mutant mobility by FRAP (Figure 4E).

FRAP curves revealed that mutant Tau proteins displayed an overall higher mobile fraction with respect to CST (85 ± 3% for P301L mutant), indicating that the P301 mutation increases Tau mobility (Figure 4E, Supplementary Figure S3, Supplementary Table S1). Remarkably, the rapid diffusion phase doubles for the CST-P301L (47 ± 4%) highlighting that the Pro to Leu mutation impacts on the balance between diffusive and binding phases. The time constants of both diffusing and bound phases are not altered, indicating that Tau molecule is not proteolytically processed, as confirmed by the western blot analysis (Supplementary Figure S3B).

We have further employed active and defective Tau mutants to investigate the conformational properties of Tau in relation to its interactions with MT. It is well known that phosphorylation of Tau affects its interaction with MTs, moreover, a fraction of CST is phosphorylated in HeLa cells (Supplementary Figure S3C, Plouffe et al., 2012). In order to prevent the phosphorylation at the AT8 epitope, that is known to occur at an early stage of the disease and to favor the phosphorylation of other pathological epitopes that are related to the MT destabilization, we generated the S199A-S202A-T205A AT8-epitope phosphorylation-defective mutant (CST-AT8mut; Supplementary Figure S3C, Braak and Braak, 1994; Braak et al., 1994; Bertrand et al., 2010; Bhaskar et al., 2010; Bibow et al., 2011b; Lippens et al., 2016). Conversely, we generated the defective Tau CST mutant ∆K280, also found in FTDP-17 patients and known to alter Tau interaction with MTs (Momeni et al., 2009; Hutton, 2000). Neither mutants showed alteration of the MT network (Supplementary Figure S3A).

The CST-AT8mut shows a FRET signal significantly higher than that of CST (Figures 4C,D), while the CST ∆K280 shows a FRET signal lower than that of CST and comparable to that of the P301L mutant.

FRAP measurements show that the FRAP recovery curves of the CST ∆K280 is very similar to that of the P301L mutant (Figure 4F and Supplementary Table S1), while that of the CST-AT8mut is significantly slower than that of CST, indicating a lower mobile fraction (54% vs. 76% overall mobile fraction, Supplementary Table S1).

Overall these results demonstrate that the FTDP-17 mutations P301L and ∆K280 consistently alter both the protein conformation and the interaction of Tau with MTs, conferring it an increased mobility. On the other hand, the phosphorylation-defective mutant AT8mut shows a greater binding to MTs, and, consistently, a higher FRET signal and a slower mobility.

## Discussion

Tau is a natively unfolded protein, highly soluble and with little secondary structure (Mandelkow and Mandelkow, 2012). As such, its conformations are largely dependent on its interactions with binding partners.

**Figure 5:**
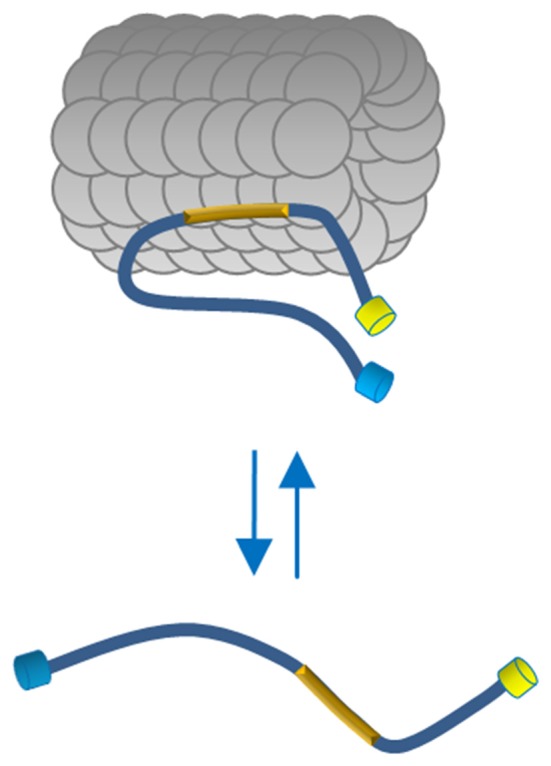
Proposed model of Tau conformation in live cells. In physiological conditions Tau binds MTs through the MTBD (yellow box) and displays a close conformation with the N and C terminus in close vicinity. The Tau molecules free in the cytosol adopt a more relaxed conformation indicating, in a cellular context, that polymerized MTs generate conformational changes of Tau.

Here we describe a FRET-based sensor that allowed us monitoring the conformational changes of full length Tau molecules in the cell context, demonstrating that the binding to MT induces a global folding of Tau, decreasing the distance between Tau N- and C-terminal domains. On the contrary, Tau molecules dislodged from MTs, did not display FRET, demonstrating, in a cellular context, that the interaction with MTs generates conformational changes of Tau (Figure 5). We operatively define the two MT-bound and MT-unbound conformations as FRET-permissive and FRET-non permissive conformations, respectively.

The presence of the two conformations, under normal conditions, is strictly dependent on Tau binding to MTs, since MT destabilization, induced by nocodazole, caused Tau detachment and a conformational change of Tau toward the FRET-non permissive state, that could be observed in real time.

The same results have been obtained by treating cells with PTX, a MT binding drug competing with Tau (Ross et al., 2004; Smoter et al., 2011). Even in this case, soluble Tau monomers in the cytoplasm displayed the FRET-non permissive conformation, confirming that it might depend intrinsically on Tau, and not on the state of the MT network.

The conformations of Tau have been extensively studied *in vitro* (Ackmann et al., 2000; Barghorn et al., 2000; Al-Bassam et al., 2002; Berriman et al., 2003) and our results confirm that, also in the cell MT-bound Tau adopts an ordered structure (Woody et al., 1983; Kar et al., 2003; Kadavath et al., 2015a,b). Our results partially agree with conclusions reached by previous *in vitro* studies employing a full length Tau in the absence of MTs (Jeganathan et al., 2012). Thus, the FRET-permissive conformation that we found, when Tau in the cell is bound to MTs, resembles the paperclip fold described by Jeganathan et al. (2012) in the test tube. They demonstrated that Tau in solution displays a global folding which brings the N-terminus in the vicinity of the C-terminus, and the C-terminus, yielding a “paperclip fold” (Jeganathan et al., 2012). This evidence was confirmed by NMR analysis (Mukrasch et al., 2009) and paramagnetic relaxation enhancement (PER) measurements *in vitro* (Bibow et al., 2011a,b).

We found that, in the cell, this “paperclip fold” (that we define as the FRET-permissive conformation) is lost when Tau is dislodged from MTs. This might appear in contrast with the paperclip fold assumed by Tau in solution, *in vitro* (Jeganathan et al., 2012), however, the crowded environment of the cell cytoplasm is very different from the test tube conditions and our result might indicate that other interacting partners and modifications could contribute to determining the loss of FRET-permissive paperclip conformation of Tau in the cellular context.

Indeed, an IDP such as Tau is likely to interact with many partners in the crowded environment of the cytoplasm, preventing Tau to assume that precise paperclip structure allowing the FRET to occur. Our measurements do not allow to gain information of the Tau conformations in these MT-unbound, FRET-non permissive conditions, apart from concluding that its N- and C-terminal domains are farther apart. In any case, this underscores the importance of developing tools such as the CST to study Tau conformational changes *in vivo*.

In the pathological context, the shift from Tau monomers bound to MTs to aggregated Tau is believed to be a multi-step process (Ross and Poirier, 2004; Kuret et al., 2005; Mandelkow and Mandelkow, 2012).

There is a long-lasting debate on which could be the initial step of MT cytoskeleton changes in damaged neurons in tauopathies. Almost all the hypotheses point out the detachment of Tau from MTs, induced by phosphorylation or by tubulin depolymerization (Bramblett et al., 1993; Yoshida and Ihara, 1993; Leroy et al., 2002; Cash et al., 2003; Jope and Johnson, 2004; Hernández et al., 2013).

Our experiments with MT depolymerizing drugs or with a MT binding competitor show that increasing the levels of soluble Tau in the cell, does not lead, *per se*, to its aggregation. Hernández et al. (2013) demonstrated that the same treatments in SH-SY5Y cells increased phosphorylation of soluble Tau at the AT8 epitope without aggregation. Thus our results suggest that the Tau FRET-permissive conformation induced by MT binding could hide Tau residues that are subjected, instead, to phosphorylation in soluble unstructured monomers.

Tau P301L mutation is the first pathological mutation identified in FTDP-17, increasing its self-aggregation (Spillantini et al., 1998a,b; Gasparini et al., 2011). P301 point mutants showed an increased interaction between Tau and free Tubulin dimers but a negligible effect on binding to stabilized MTs *in vitro* (Elbaum-Garfinkle et al., 2014). CST allowed to conclude that P301L Tau mutant does not form fluorescent aggregates in the cell, but shows an impaired decoration of MT network, in agreement with previous studies (Hasegawa et al., 1998; Dayanandan et al., 1999).

Our analysis provides a structural basis to this finding, showing that P301 mutation alters Tau protein conformation, since its FRET signal, when bound to MT, is lower. This could be due to Tau P301L having a more opened structure when bound to MTs, compared to wt Tau.

Moreover, CST-P301L highlighted an increase of cytoplasmic free Tau, with a doubling of the diffusive fraction, from 27% to 48%. The FRET signal measured is normalized for the intensity of the donor and the acceptor (NFRET) thus excluding a contribution from different amount of fluorophores on MTs.

Thus, the distinct Tau conformation of MT-bound Tau, caused by the P301 mutation, could be responsible for its weaker interaction with MTs.

The other mutant (∆K280) involved in FTDP-17 pathology showed the same behavior in the impaired interplay between Tau and MTs, as P301L, suggesting that different point mutations in the MTBD are similarly deleterious.

This study brought the first Tau probe that allows the investigation of the full length tau conformations in live cells in the presence of mutations and in a physiological context.

However, as all FRET-based systems, also the CST could be affected by intrinsic limitations. In particular, we point to unpredictable reorientational dynamics of the donor/acceptor pair. Indeed, we cannot exclude a partial contribution of this phenomenon in both FRET-permissive and -nonpermissive conformations, also considering that Tau flexibility/rigidity, as all intrinsically disordered proteins, may change between the free and bound states. However, the loss or the reduction of FRET in different unrelated conditions, such as cell treatments (PTX or Nocodazole) and pathological mutations, indicates that the possible contribution of the dipole reorientation might be very low. Moreover, dos Remedios and Moens (1995) argued that the orientation parameter is not an important issue in FRET protein probes. In general the FRET limitation is the inseparability of structural and dynamical terms. Moreover, it could be argued that crowding on the MT might affect the FRET signal. However, we demonstrated that the FRET signal on MT is intramolecular and not intermolecular, so it is independent on Tau crowding. We cannot exclude that the FRET signal might be influenced by changes in the crowding of other proteins on the MT.

The instability of Tau/MT interplay revealed by FRAP, could represent an early step of the pathology, determining a reduced number of Tau molecules available for binding and stabilizing MTs and an increased amount of soluble mutant molecules sensitive to pathological post-translational modifications.

Based on these data, we hypothesize that the FTDP-17 Tau mutations weaken the interaction of Tau with cellular MTs, thus shifting the equilibrium towards the soluble pool. One might also envisage that the mutations may affect the Tau conformation on MTs, in such a way that the flexibility of the N- and C- termini is affected. As for the phospo-Tau AT8 mutant, we likewise suggest that the observed behavior is explained by shifting its equilibrium towards the MT-bound state, assuming a more closed conformation.

The properties of CST make it amenable to be used for a versatile set of different large-scale cell screenings. Thus, cell models allowing to monitor Tau assembly have been recently established (Kfoury et al., 2012; Tak et al., 2013; Holmes and Diamond, 2014; Holmes et al., 2014; Sanders et al., 2014; Vasconcelos et al., 2016; McEwan et al., 2017). However, the latter is based on mutated aggregation-prone full length Tau to increase the sensitivity of the readout, that does not mimic a physiological state of Tau, while the others are based on Tau RD fragments, that do not allow to investigate the misfolding process of full length native protein nor to take into account cellular cofactors modulating Tau conformation outside the RD domain.

Instead, CST is a powerful tool, to image the properties of the human full length Tau protein in living cells, and provides quantitative and qualitative readouts related to the physiological interactions of wt Tau with MTs in cells and to its pathological aggregation in response to synthetic seeds. There is a big interest in developing compounds or treatments to modulate the cellular properties of Tau in a therapeutic perspective (Himmelstein et al., 2012; Iqbal et al., 2014). It is envisaged that the CST can form the basis for cellular assays to screen for compounds lowering the levels of Tau, shifting its distribution from the soluble to the MT-bound compartments or preventing its aggregation. Along these lines, CST could also be used in an *in vivo* setting, such as in zebrafish or in mice, to advance our knowledge on Tau biology or on Tau as a therapeutic target for neurodegenerative diseases.

## Author Contributions

CDP and VQ contributed equally. CDP, VQ and AC designed experimentation and wrote the manuscript. CDP, VQ, GS and MR performed the experiments, collected and analyzed the data. BK and MN provided reagents and contributed with discussion and correction of the manuscript.

## Conflict of Interest Statement

The authors declare that the research was conducted in the absence of any commercial or financial relationships that could be construed as a potential conflict of interest. The reviewer NRG and handling Editor declared their shared affiliation, and the handling Editor states that the process nevertheless met the standards of a fair and objective review.

## Supplementary Material

FIGURE S1 Box plot describing NFRET values for FRET negative control (cells expressing the donor and the acceptor by transfecting separated plasmids pECFP and pEYFP) and FRET positive control (cells expressing the donor and the acceptor fused into a chimeric construct ECFP-EYFP) Box spans the standard error of the mean (black square) while whiskers indicate the standard deviation (***p* < 0.001 ANOVA one-way test). FIGURE S2 **(A)** Western blot analysis of CST reporter cells treated with nocodazole. **(B)** Imaging of CST reporter cells untreated or treated with Paclitaxel or Nocodazole. Cells have been subjected to immunofluorescence to label Tubulin (red); donor channel (blue), acceptor channel (yellow). White scale bar = 10 μm. **(C)** Western blot analysis of cells treated with Paclitaxel. **(D)** Residual recovery of CST, in untreated cells and in cells treated with Noc or PTX, between experimental data and either a mono-exponential equation (gray curve) or a bi-exponential equation (black curve). FIGURE S3 **(A)** Immunofluorescence against Tubulin (red) of reporter cells. For each image a magnification is reported. **(B)** Western blot analysis of cells transfected with mutant CST constructs. **(C)** Western blot analysis of phosphorylation profile at the AT8 epitope of Tau in wt CST and CST-AT8mut in Hela reporter cells. **(D)** Image analysis by IMARIS of the Tau fluorescent network (yellow, left panel) and identification and analysis of the fluorescent filaments and the crossover points (right panel). **(E)** Residual recovery of CST-P301L between experimental data and either a mono-exponential equation (red curve) or a bi-exponential equation (black curve). TABLE S1 Mobility and binding characteristics in FRAP experiments. Relative recovery curves were fitted with a double exponential to obtain the mobility parameters and the mobile fraction (Mob_calc_) in presence or not of microtubule modifications by drugs or in presence of Tau point mutations. Values represent means ± SEM. The condition “Noc treatment” corresponds to cells treated with 1 μM nocodazole (30 min before and maintained during FRAP experiment) to disassemble microtubules. The condition “PTX treatment” corresponds to cells treated with 1 μM Paclitaxel (10 min before and maintained during FRAP experiment) to stabilize microtubules and detach Tau from MTs. A_1_ corresponds to the percentage of mobile molecules in the diffusing soluble phase, A_2_ corresponds to the percentage of mobile molecules in the MT-binding phase. τ1 and τ2 are the associated time constants.

## Abbreviations

AD: Alzheimer’s Disease
CST: Conformational Sensitive Tau biosensor
FRAP: Fluorescence Recovery After Photobleaching
FRET: Fluorescent Resonance Energy Transfer
FTDP-17: Frontotemporal Dementia Linked to chromosome 17
MTBD: Microtubule Binding Domain
MTs: microtubules
NFT: Neurofibrillary tangle
Noc: Nocodazole
PTX: Paclitaxel.
