# Supplementary material for: The Distance between N and C Termini of Tau and of FTDP-17 Mutants Is Modulated by Microtubule Interactions in Living Cells
Source: Front Mol Neurosci. 2017 Jun 30;10:210. doi: 10.3389/fnmol.2017.00210 (PMC5492851; doi:10.3389/fnmol.2017.00210)
Supplement: Supplementary file 1 [file Presentation_1.pdf]

Supplementary Figure 1

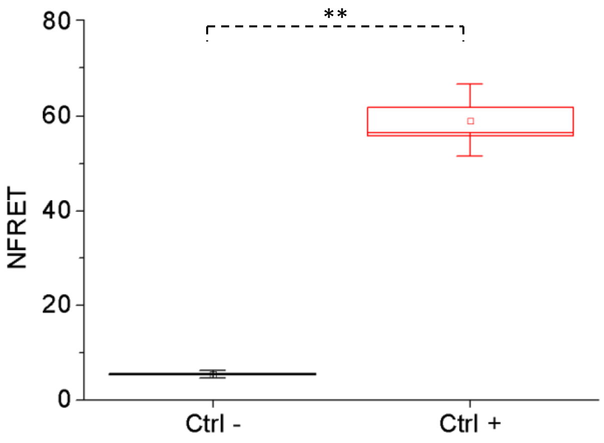

Supplementary Figure 2

A

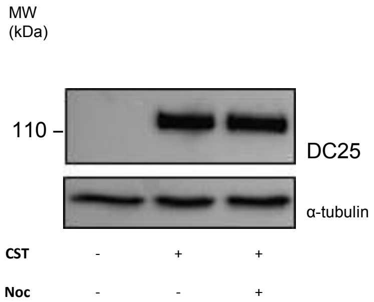

B

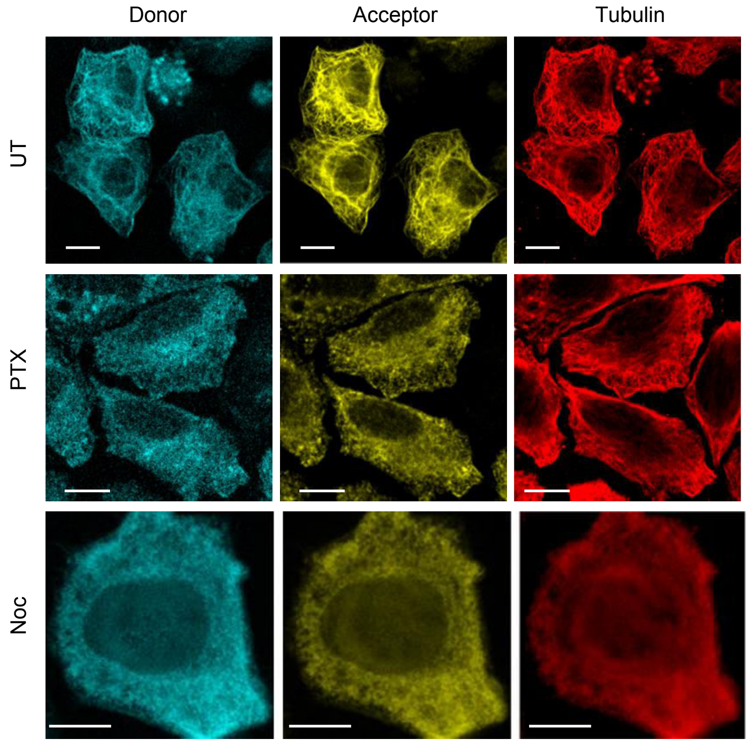

C

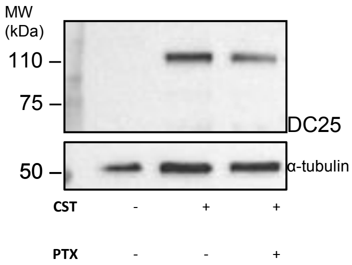

D

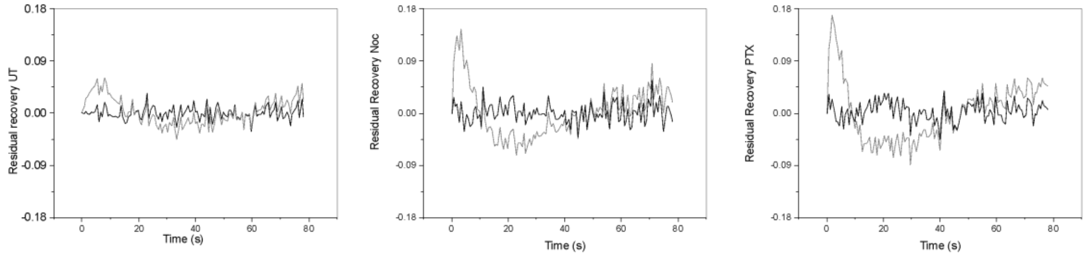

Supplementary Figure 3

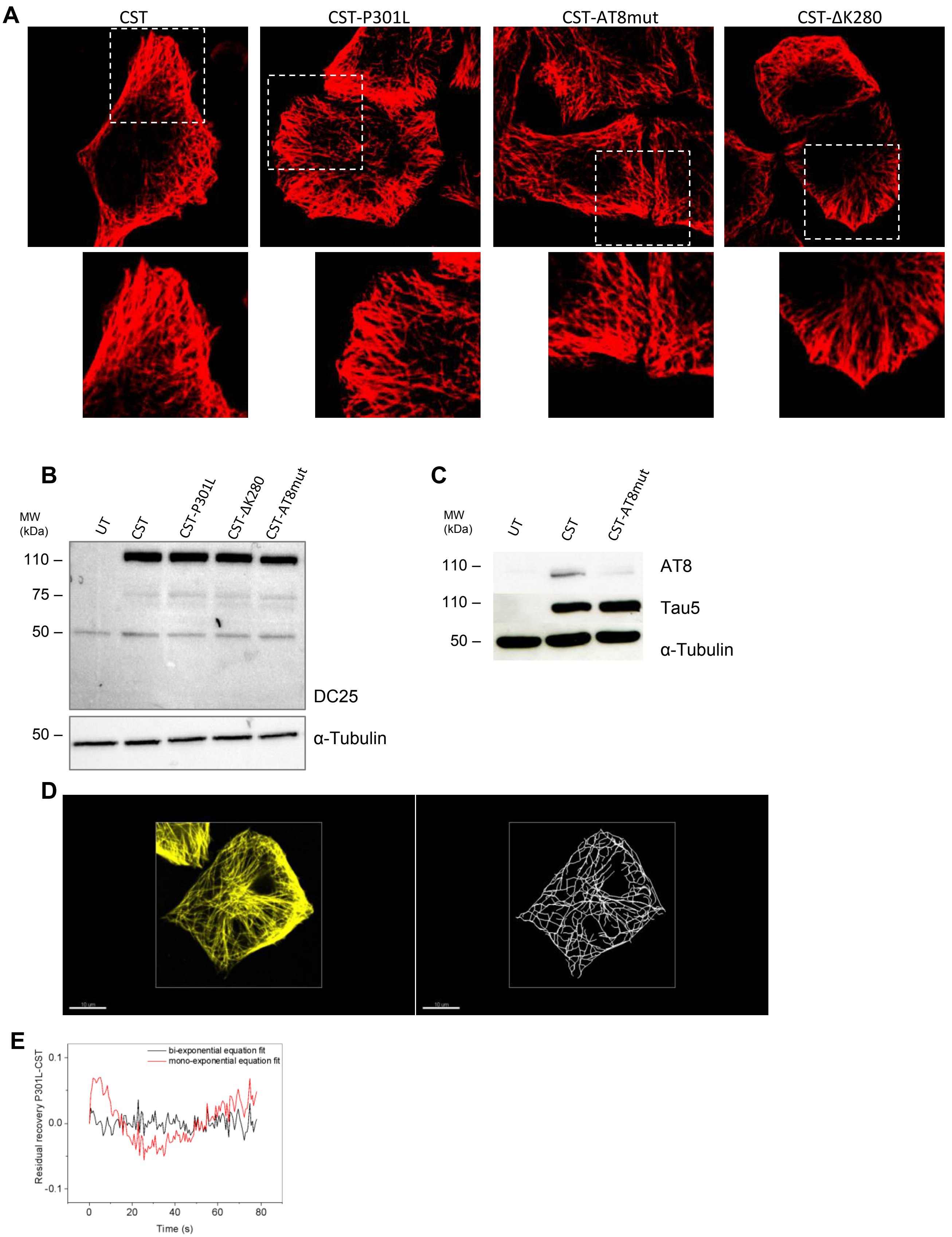

	Mob_{calc} (%)	A_1(%)	A_2(%)	τ_1(s)	τ_2(s)
**CST**	76±3	27.8±4.1	72±5	4.99±0.6	35.2±2.8
**NOC treatment**	91±4	53±5	47±4	3.3±0.5	28±2
**PTX treatment**	93±3	55±3	45±3	2.9±0.6	29.5±1.5
**CST-P301L**	85±3	47.4±4.1	52.6±3.4	4.5±0.3	33.2±2.8
**CST-ΔK280**	89±3	47±3	53±3	3.6±0.1	29±1
**CST-AT8mut**	54±1	7.5±0.5	92.5±1.8	2.8±0.2	39.7±0.9

Supplementary Table 1
